# The Race of Man; a Fragment

**Published:** 1851-01

**Authors:** 


*The Race of Man; A Fragment.* By Robert Knox, M. D., Lecturer on Anatomy, and corresponding member of the National Academy of Medicine of France. Philadelphia : Lea & Blanchard. 1850. 12 mo. pp. 323.

We have dipped a little into this work, but we do not profess any competency to judge of its merits, and it is so foreign to subjects most congenial to our taste, that we do not expect to give it a careful perusal.

The author is known as the English Editor of Quain's Anatomy. The proposition which it is his aim to unfold and establish is, that in the character of countries, and individuals, everything is due to *race*; literature, science, art, in a word, civilization depending on it. He appears to disclaim all belief in revealed truth, and doubts whether Christianity has done much for civilization!

For sale by Phinney, & Co.

